# Supplementary material for: Assessment of missed opportunities for vaccination in Kenyan health facilities, 2016
Source: PLoS One. 2020 Aug 20;15(8):e0237913. doi: 10.1371/journal.pone.0237913 (PMC7440639; doi:10.1371/journal.pone.0237913)
Supplement: S2 File — (PDF) [file pone.0237913.s002.pdf]

ANNEX 2: HEALTH FACILITY EXIT SURVEY

Good morning/afternoon. My name is ______________________________ and I am working on a programme assessment of the vaccination of children in this locality. Our goal is to help improve the vaccination programme in general. I would like to respectfully ask for your help in answering the questions in this assessment. I know you are busy, so we will interview you for only a few minutes. Your participation is voluntary and anonymous. Would you be willing to answer these questions? Thank you very much.*

Questionnaire Serial Number ☐☐☐ THIS IS PRE-ASSIGNED CENTRALLY

Date of interview
Day |__|__| Month |___|___ Year |___|___|

Interview start time:
Hour |__|__| Minutes |___|___|

GEOGRAPHICAL LOCATION

Name of interviewer: __________________ Supervisor: _______________
Name of health facility: __________________
Sub-county ________________________________ County _________________

A. Classification of this health facility

1. Public/Government service ☐
2. Private ☐
3. Non-Profit ☐
4. Faith-based organization ☐
5. Other ☐ Specify: _____________________________

B. Type of health facility

1. Hospital ☐
2. Clinic ☐
3. Health center ☐
4. Health Post ☐

C. Filter

The child appears to be <24 months
1. Yes ☐ ➡ CONTINUE (RECORD RESPONSE IN TRACKING FORM PLEASE)
2. No ☐ ➡ **THANK THE PERSON AND RECORD THE ENCOUNTER IN TRACKING FORM**

SECTION 1: DATA ON THE CHILD
(IN CASE OF MORE THAN ONE CHILD, CHOOSE THE YOUNGEST CHILD)

1. Date of birth Day |___|___| Month |___|___| Year |___|___|
2. Sex or Gender of child 1. Male ☐ 2. Female ☐

3. Why did you bring the child to this health care facility today? **(DO NOT READ OUT THE CHOICES)**
   1. For medical consultation (child is sick) ☐
   2. For vaccination ☐
   3. Healthy child visit or growth/development check-up ☐
   4. Child is only accompanying (not for treatment, vaccination) ☐
   5. Hospitalization (child was admitted or is still on admission) ☐
   6. Other ☐ Specify: ____________________

SECTION 2: DATA ON THE CHILD'S CAREGIVER, PARENT/GUARDIAN

5. What is your relationship to the child?

1. Mother ☐
2. Father ☐
3. Grandparent ☐
4. Uncle/aunt ☐
5. Brother/sister ☐
6. Other ☐ Specify: ____________________

6. Can you read and write?

1. Yes ☐
2. No ☐

7. Level of formal education

1. No formal education ☐
2. Did not complete primary (less than 6 years) ☐
3. Completed primary ☐
4. Completed secondary school ☐
5. More than secondary ☐

8. What do you do for a living?

1. Housewife (work is housekeeping) ☐
2. Employee or laborer ☐
3. Farming ☐
4. Self-employed ☐
5. Boss or employer ☐
6. Other ☐ Specify: ____________________

9. By what means of transportation do you usually come to this facility?

1. Walk ☐
2. Bicycle ☐
3. Motorcycle ☐
4. Car ☐
5. Bus ☐
6. Ox carts ☐
7. Other ☐ Specify: ____________________

10. How long does it take you to get here? _____Hours _____Minutes

11. Have you heard or seen messages on vaccination in the last one month?

1. Yes ☐ ➡ CONTINUE WITH QUESTION 12
2. No ☐ ➡ SKIP TO QUESTION 13

12. Where/how did you hear or see the message? **(DO NOT READ OUT THE OPTIONS. CHECK ALL THAT APPLY)**

1. Radio ☐
2. Television ☐
3. Newspaper ☐
4. Health facility ☐
5. Telephone message ☐
6. Facebook or internet ☐

7. Children's school ☐
8. Place of worship ☐
9. During home visit by health workers/health outreaches ☐
10. Community meetings ☐
11. Other ☐ Specify: ____________________

13. Do you feel that you know the vaccines your child needs and when they should be given?

1. Yes ☐
2. No ☐
3. Not sure ☐

14. Has this child ever been vaccinated?

1. Yes ☐
2. No ☐

15. Have you ever requested vaccination service for this child and been refused?

1. Yes ☐
2. No ☐ ➡ SKIP TO QUESTION 17

16. If so, why didn't they vaccinate the child?

1. The doctor or nurse said it couldn't be done because the child was sick ☐
2. There were no vaccines, or there were no syringes or some other supply needed for vaccination ☐
3. It was not a vaccination day ☐
4. The vaccination area was closed ☐
5. The person in charge of vaccination was not there ☐
6. We didn't have the vaccination card/passport ☐
7. The hours for vaccination are limited ☐
8. Other ☐ Specify: ____________________

17. In your home, who makes the decision to vaccinate the children

1. Father ☐
2. Mother ☐
3. Other relatives ☐
4. Consensus of father and mother ☐
5. Other ☐ Specify: ____________________

SECTION 3: USE OF VACCINATION CARD/HEALTH PASSPORT AND INFORMATION ON VACCINES ADMINISTERED

18. Does your child have a vaccination card/health passport?

1. Yes, and I have it with me ☐ ➡ GO TO QUESTION 20
2. Yes, but I do not have it with me ☐ ➡ GO TO QUESTION 19a with Annex 5
3. No ☐ ➡ GO TO QUESTION 19b with Annex 5

19a. Could you tell us why you do not have the vaccination card/health passport with you today?

1. It is at the nursery school/day care center ☐
2. I left it at home (because I forgot to bring it) ☐
3. I left it home (because I didn't know it was important to bing it along) ☐
4. I lost it ☐ ➡ SKIP TO QUESTION 22

5. The card/health passport has been damaged ☐
6. I have never been given one ☐ ➡ SKIP TO QUESTION 23
7. Because vaccination was not the reason for this visit ☐
8. Other ☐ Specify: ____________________

19b. Why don't you have a vaccination passport?

1. I lost it ☐ ➡ SKIP TO QUESTION 22
2. I have never been given one ☐ ➡ SKIP TO QUESTION 23
3. I don't know ☐
4. Other ☐ Specify: ____________________

Whenever the vaccination card or health passport is not with the caregiver today, request to complete the information in Annex 5 (Health Facility Register Follow-Up Form). Assure them that this information will only be used to match the records in the health facility register.

At the end of all the interviews, use the information from Annex 5 to complete the table below.

Remember to ALSO take a picture of the relevant pages/lines of the register. ➡ **GO TO QUESTION 21**

20. Request and examine the child's vaccination card/health passport or temporary vaccination document to fill out the following table.

Remember to take pictures of all the completed pages on the health passport/card

Date Administered, as written on the vaccination card or health passport

**Vaccines**	***Dose 0***	***Dose 1***	***Dose 2***	***Dose 3***
**BCG**		___/___/___		
**Oral Polio**	___/___/___	___/___/___	___/___/___	___/___/___
**IPV**		___/___/___		
**DPT-HepB-Hib**		___/___/___	___/___/___	___/___/___
**Rotavirus**		___/___/___	___/___/___	___/___/___
**Pneumo (PCV)**		___/___/___	___/___/___	___/___/___
**Measles or MR**		___/___/___	___/___/___	
**Yellow Fever**		___/___/___		

20b. Please review the entire maternal and child health booklet and indicate which recording areas are available and which ones have been filled. A recording area is considered filled or marked if ANY **deliberate** mark or information is included. If it is unclear whether there are deliberate markings or recorded information, perhaps due to damage to the document, then mark that you are unsure. **CHECK ALL THAT APPLY**

	**Recording area available?**		➡	**Recording area marked?**	
	No	***Yes***		***Yes***	***Unsure***
a) Child background information	☐	☐		☐	☐
b) Vaccination history	☐	☐		☐	☐
c) Vitamin A	☐	☐		☐	☐
d) Growth monitoring chart	☐	☐		☐	☐
e) Early eye or vision problems	☐	☐		☐	☐
f) Newborn child delivery	☐	☐		☐	☐
g) Not applicable (Document is not a Kenya MCH booklet)	☐				

21. Have you ever lost a vaccination card/health passport for this child?

1. Yes ☐
2. No ☐ ➡ **SKIP TO QUESTION 23**

22. Did you encounter difficulty getting it replaced?

1. Yes ☐
2. No ☐

23. Could you tell me what purpose the vaccination card/health passport serves?

1. To know what vaccines the child has had and which ones are missing ☐
2. Other ☐ Specify: ____________________
3. Don't Know/No Response ☐

SECTION 4: TODAY'S VACCINATION

24. During today's visit, did the personnel/staff ask you for the child's vaccination card/health passport?

1. Yes ☐ ➡ SKIP TO QUESTION 27
2. No ☐ ➡ SKIP TO QUESTION 25

25. If No, did they ask for the vaccination status of the child?

1. Yes ☐
2. No ☐

26. Is this child up-to-date for all vaccines for which he/she is eligible?

1. Yes ☐
2. No ☐
3. I don't know ☐

27. Was your child vaccinated here today?

1. Yes ☐ ➡ **SKIP TO QUESTION 30**
2. No ☐

---

28. Why didn't they vaccinate the child today? (Please DO NOT read the options)

FIRST LISTEN TO THE REASONS GIVEN BY THE CAREGIVER AND THEN TRY TO CHOOSE THE RIGHT OPTION FROM BLOCK A, B OR C BELOW

BLOCK A: REASONS RELATED TO THE HEALTH WORKERS

1. The doctor/nurse said that the child was not eligible for vaccination today ☐
2. The health worker who saw us did not tell me about vaccinating the child today ☐
3. The doctor/nurse said that the child could not be vaccinated because he/she was sick ☐

IF BECAUSE OF ILLNESS, WHAT TYPE OF DISEASE OR TREATMENT DID THE CHILD RECEIVE TODAY?

1. Minor illnesses such as mild fever, cold, cough, or diahrroea ☐
2. Major illnesses requiring admission, such as severe pneumonia or severe malaria ☐
3. Other illnesses such as intestinal parasitosis, malnutrition, anaemia, dehydration, urinary tract infection ☐
4. Child is taking medications: ☐ Write down generic name ______________
5. HIV or AIDS ☐
6. Other ☐ Specify: ____________________

Interviewer: IF THE REASONS CITED BY THE INTERVIEWEE REFER TO BLOCK A ABOVE, ➡ SKIP TO QUESTION 40

BLOCK B: REASONS RELATED TO THE CAREGIVER

1. The last time the child was vaccinated, he/she got sick or had a reaction. ☐
2. My religion doesn't permit vaccination or I don't believe in vaccines ☐
3. Vaccination was not the purpose of this visit ☐
4. This child is already fully vaccinated for his/her age ☐
5.I don't trust the health workers/the vaccines in this health facility ☐
6. I forgot to take my child to the vaccination area ☐
7. I didn't have time today to wait for vaccination ☐
8. Other ☐ Specify: ____________________

Interviewer: IF THE REASONS CITED BY THE INTERVIEWEE REFER TO BLOCK B ABOVE, ➡ SKIP TO QUESTION 40

BLOCK C: REASONS RELATED TO THE HEALTH FACILITY (LOGISTICS & ORGANIZATION)

1. There were no vaccines in the health facility today ☐
2. There were no syringes or other vaccination supplies ☐
3. Today is not a vaccination day in this health facility ☐
4. The vaccination area was closed ☐
5. The person in charge of vaccinations was not there ☐
6. There would have been a long wait ☐
7. The staff treated us badly ☐
8. Other ☐ Specify: ____________________

Interviewer: IF THE REASONS CITED BY THE INTERVIEWEE REFER TO BLOCK C ABOVE, ➡ SKIP TO QUESTION 40

---

29. If your child was eligible for vaccination but was not vaccinated today, did the health worker refer you to or inform you where you can receive the missing vaccine doses?

1. Yes ☐
2. No ☐

SECTION 5: QUALITY OF THE VACCINATION SERVICE

30. How long did you wait today for your child to be vaccinated? Hours: _____ Minutes: ______

31. Did they tell you today what vaccines they gave the child?

1. Yes ☐
2. No ☐

32. Today, did they tell you the date of the next vaccination appointment?

1. Yes ☐
2. No ☐

33. Today, did they write down for you the date of the next vaccination appointment?

1. Yes ☐
2. No ☐

34. Did you receive information today on the reactions or side effects that can occur following vaccination?

1. Yes ☐
2. No ☐ ➡ **SKIP TO QUESTION 37**

35. If so, what did they mention? (choose all that apply)

☐ Pain at injection site
☐ Fever
☐ Rash
☐ Diarrhea
☐ Vomiting
☐ Other Specify: ___________________

36. Did you receive information today on what you should do if the child has reactions or side effects to the vaccines?

1. Yes ☐
2. No ☐

37. Are you satisfied with the service provided today?

1. Yes ☐
2. No ☐ ➡ **SKIP TO QUESTION 39**

38. Why were you satisfied with the service?

1. Immediate attention ☐ ➡ **SKIP TO QUESTION 40**
2. Friendly treatment by staff ☐ ➡ **SKIP TO QUESTION 40**
3. No charge for service ☐ ➡ **SKIP TO QUESTION 40**
4. Other ☐ Specify: __________ ➡ **SKIP TO QUESTION 40**

39. Why were you NOT satisfied?

1. Had to wait a long time ☐
2. The staff was discourteous ☐
3. The language that the health workers use is not clear ☐
4. They did not explain what vaccines they had given the child ☐
5. Other ☐ Specify: __________

40a. Have you ever been asked to pay for vaccines given to a child?

1. Yes ☐
2. No ☐

40b. What type of health facility asked you to pay?

1. Public ☐
2. Private ☐
3. Don't know ☐

41a. Have you ever been asked to pay for a health card/passport for a child?

1. Yes ☐
2. No ☐

41b. What type of health facility asked you to pay?

1. Public ☐
2. Private ☐
3. Don't know ☐

SECTION 6: REASONS TO VACCINATE CHILDREN

42. Could you tell me the purpose of vaccines? (CHECK ALL THAT APPLY) **Please DO NOT read out the options**

1. To prevent diseases ☐
2. So children will grow up healthy ☐
3. To cure/heal diseases ☐
4. They don't do any good ☐
5. Not sure what they are for ☐
6. Other ☐ Specify: __________

43. Do you think your child could get diseases if you don't vaccinate him/her?

1. Yes ☐
2. No ☐

44. What suggestions do you have to improve vaccination services? **(CHECK ALL THAT APPLY)**

1. There should be more vaccination personnel ☐
2. There should be less of a wait ☐
3. Hours and days when vaccinations are available should not be limited ☐
4. Vaccination should remain free ☐
5. The treatment of the public, and of the children being vaccinated, should be friendlier ☐
6. The health center should always have vaccines ☐
7. They should provide information on the vaccines that are being given, on the diseases that they prevent, and on the reactions that they produce. ☐
8. Other ☐ Specify: ___________________
9. None ☐
10. Don't know ☐

Interviewer: Thank the interviewee and note the time when the interview was concluded. Read the following statement:

"Remember that vaccination is a right for all people. Demand this right and remember to bring your child's vaccination card to the health facility each time you visit the centre for any reason."

Interviewer's remarks: ____

____

____

Supervisor: Please check the completed form for accuracy and completeness

1. Form is complete and accurate (skip patterns adequately observed) ☐
2. There are no errors or inconsistencies on the form ☐

Supervisor's remarks: ____

____

____

Supervisor's full name: ____

Supervisor's signature: ____
