# Supplementary material for: Assessment of missed opportunities for vaccination in Kenyan health facilities, 2016
Source: PLoS One. 2020 Aug 20;15(8):e0237913. doi: 10.1371/journal.pone.0237913 (PMC7440639; doi:10.1371/journal.pone.0237913)
Supplement: S3 File — (PDF) [file pone.0237913.s003.pdf]

ANNEX 3: HEALTH WORKER QUESTIONNAIRE

The Ministry of Health, in collaboration with the World Health Organization and UNICEF wishes to strengthen the technical skills of all health workers, especially those who provide immunization services. This questionnaire has been designed to identify future training topics in immunization for all health workers. Your collaboration is greatly appreciated. Your name is not included in this questionnaire and your participation is voluntary.

If you decide to participate, please use a pen to mark answers that in your opinion respond appropriately to the question or problem presented. Responses will not serve as the basis for any evaluation of your professional abilities. ***Read each section of the questionnaire carefully, and please do not leave any questions blank.***

Questionnaire Serial Number ☐☐☐

Date of interview Day |__|__| Month |___|___| Year |___|___|

GEOGRAPHICAL LOCATION

Name of interviewer: ___________________ Supervisor: ________________
Name of health facility ___________________
Sub-county _____________________________________ County __________________

A. Classification of this health facility

1. Public/Government service ☐
2. Private ☐
3. Non-Profit ☐
4. Faith-based organization ☐
5. Other ☐ Specify: _______________________________

B. Type of health facility

1. Hospital ☐
2. Clinic ☐
3. Health center ☐
4. Health Post ☐

I. BACKGROUND INFORMATION

PLEASE MARK THE CORRECT ANSWER IN THE FOLLOWING SECTION:

1. Gender or sex ☐ 1. Male ☐ 2. Female

2. Age ☐ 1. Under 20 years ☐ 2. 20-29 ☐ 3. 30-39 ☐ 4. 40-49 ☐ 5. 40-49 ☐ 6. 50 or over

3. What is your professional training?

1 Doctor ☐
2 Nurse ☐
3 Clinical Officer ☐
4 Public Health Officer ☐
5 Other ☐ Specify: _____________________

4. Area (or department) in which you work

1. In-patient Department (in the admission wards) ☐
2. General Out-Patient (OPD) ☐
3. Emmergency Department ☐
4. Immunization, preventive medicine and epidemiology ☐
5. Nutrition ☐
6. IMCI (Integrated Management of Childhood Illnesses) ☐
7. Dental/Oral Unit ☐
8. Family Planning and STI ☐
9. Ante-Natal Clinic (ANC) ☐
10. Other ☐ Specify: ____________________

5. For how long have you been in this post? |___|___| years |___|___| months

6. During your basic training in nursing, midwifery or medical school, were you trained in the control of vaccine-preventable disease?

1. Yes ☐
2. No ☐

7. Have you ever received training or participated in courses on vaccination or control of vaccine-preventable diseases?

1. Yes ☐
2. No ☐ ➡ **SKIP TO QUESTION 9**

8. If YES, when were you last trained?

1. <1 year ago ☐
2. 1-2 years ago ☐
3. 2-3 years ago ☐
4. >4 years ago ☐

II. KNOWLEDGE OF VACCINATION

FOR QUESTIONS 9 - 11, PLEASE CHECK ALL CORRECT OPTIONS

9. Vaccines that healthy children should receive include: (PLEASE CHECK ALL THAT APPLY)

1. BCG ☐
2. Measles ☐
3. Pentavalent ☐
4. Polio vaccine ☐
5. Rotavirus vaccine ☐
6. Pneumo (PCV) ☐

10. Contraindications against being vaccinated with polio vaccine include:

1. Breastfeeding ☐
2. Axillary or rectal temperature of 37.5 C ☐
3. Mild malnutrition ☐
4. Mild diarrhea ☐
5. None of the above ☐

11. Please match the vaccines listed below with the age at which they should be administered. **Please write in the blank column** of the first box the number (e.g. 5) that corresponds to the correct answer in the second box

		Blank column
A	Pentavalent	
B	IPV	
C	BCG	
D	OPV	
E	1st dose of Measles or MR vaccine	

1	At birth, and 6, 10 and 14 weeks
2	6, 10, 14 weeks
3	9 months
4	14 weeks
5	At birth

FOR QUESTION 12, PLEASE MARK ONLY ONE CORRECT ANSWER

12. Vaccine-preventable diseases (VPDs) in the process of eradication or elimination include:

Mark only one response:

1. TB ☐
2. Whooping cough ☐
3. Poliomyelitis and measles ☐
4. Diarrhea ☐
5. None of the above ☐

FOR QUESTION 13, PLEASE CHECK ALL CORRECT OPTIONS

13. Absolute contradications against ANY vaccine include:

1. Local reaction to previous dose ☐
2. Light fever ☐
3. Seizures under medical treatment ☐
4. Pneumonia or other serious diseases ☐
5. None of the above ☐

III. ATTITUDES

FOR QUESTIONS 14-15, PLEASE MARK ONLY ONE CORRECT ANSWER.

14. From day to day, who should evaluate the vaccination status of children, review vaccination cards/health passports, and ensure that children are up to date according to the national schedule?

1. The child's parents ☐
2. The health worker responsible for immunization ☐
3. Physicians in external consultations, inpatient services, and emergency rooms ☐
4. All of the above ☐

15. In which of the following situations should you inquire about the doses that children have received and those that are missing according to their age?

1. During a child's wellness visit ☐
2. Consultation for any illness ☐
3. When a child is accompanying a caregiver during a pre-natal check-up ☐
4. When a child is accompanying a caregiver visiting a health care facility for any reason ☐
5. All of the above ☐

FOR QUESTION 16, PLEASE CHECK ALL CORRECT OPTIONS

16. Why do you think that some children are not up to date on their vaccination?
    1. Parents' negative beliefs related to vaccination ☐
    2. Hours of vaccination incompatible with parents' busy lives ☐
    3. Physicians, nurses, and health workers do not ask about children's vaccination schedules ☐
    4. Physicians, nurses, and health workers do not review children's vaccination records ☐
    5. False contraindications for vaccination by health workers ☐
    6. Distance from vaccination site ☐
    7. All of the above ☐

17. Do you believe that the vaccines administered in private practice vary in quality from those provided by the Ministry of Health?
    1. Yes ☐
    2. No ☐
    3. Don't know ☐

18. Please explain your response in Question 17: ____

IV. PRACTICES

IV.I DECISION MAKING IN DAILY PRACTICE

FOR QUESTIONS 19-32, MARK THE ONE CORRECT ANSWER

19. A female infant comes to the clinic today. She is aged 3 months. She has a documented history of one dose of BCG and one of OPV0, both administered at birth. The mother seeks service to assess the child's growth and development. What vaccines would you give the child today?
    1. None ☐
    2. Only Polio ☐
    3. Only Pentavalent ☐
    4. Measles vaccine ☐
    5. Polio, Pentavalent, Rotavirus, and PCV ☐
    6. Don't know ☐

20. Female infant aged 6 months with documented history of one dose of BCG, two doses of pentavalent, and two doses of polio vaccine. The last doses of vaccines were administered when the child was 4 months old. According to the mother, the child experienced fever and seizure one month ago and is now receiving medical treatment. Following EPI guidelines, what vaccines would you give her?
    1. I would not vaccinate her ☐
    2. Only polio vaccine and I would refer her to a specialist ☐
    3. DT5 ☐
    4. Polio and pentavalent ☐
    5. Only measles vaccine ☐
    6. Don't know ☐

21. Do you work in the area of immunization or provide vaccines as part of your job?
    1. Yes ☐ CONTINUE TO NEXT QUESTION/SECTION
    2. No ☐ SKIP TO END, "ADDITIONAL COMMENTS"

NOTE: If you work in the area of immunization or provide vaccines as part of your job, please continue to Q21.
If you work in other departments, STOP HERE and thank you for your time.

IV. PRACTICES

IV.2 IMMUNIZATION PRACTICES AND DECISION MAKING

[THIS SECTION IS ONLY FOR ALL HEALTH CARE PROFESSIONALS WHO ADMINISTER VACCINES]

22. Under what circumstances would you tell the parent what vaccines you are administering AND provide advice regarding what to do in case the child experiences an adverse reaction following immunization?
    1. Only if the vaccine administered could produce a severe reaction ☐
    2. Only when the parent or guardian requests this information ☐
    3. Never, since this information can be counterproductive and discourage participation in the immunization programme ☐
    4. Always, regardless of the vaccine used and type of reaction that might be expected ☐
    5. The probability that an adverse event related to vaccination is so low that I would rarely have to provide this information ☐

23. Today, you vaccinate a female child aged 2 months with the first doses of pentavalent/DTP and polio vaccines. After telling her parents which vaccines she received, what other information and recommendations would you provide her caregivers? (choose all that apply)
    1. The child may experience a bit of fever, diarrhea, or discomfort following vaccination ☐
    2. The symptoms above generally do not require treatment; however, in the case of fever, the child should be lightly dressed and should NEVER stop breastfeeding ☐
    3. The parent should return to the health center if these symptoms persist so that the child may be seen by a doctor ☐
    4. None of the above ☐

24. What should be done if you notice that there are children with delayed or missed vaccines in the nominal registeries? (choose all that apply)
    1. Make a weekly list of children with incomplete schedules ☐
    2. Contact parents or guardians by telephone, email, or any other means of communication to remind them to vaccinate their children ☐
    3. Make home visits to encourage the family to complete the child's vaccination schedule and administer missing doses while there ☐
    4. None of the above ☐

25. What could be done to follow up on vaccination of children after hospitalization or outpatient treatment for a chronic condition? (choose all that apply)
    1. Coordinate with clinical areas, inpatient and emergency departments in hospitals, so that they can review the child's vaccination card/health passport ☐
    2. Send patients whose physicians consider them eligible for vaccination to the immunization department so that they can be vaccinated before leaving the hospital ☐
    3. In hospitals, a health worker in the immunization department could visit inpatient departments to review to review the medical records of children who will be discharged that day, thereby identifying children to start or complete the vaccination schedule ☐
    5. None of the above ☐

26. At 8:00 am, you prepare a vaccination cold box for the morning shift at the health facility. You place two vials of 10 doses of measles vaccine in the cold boxes. At 3:00 pm, a mother requests that her 14 month old child receive one dose of measles vaccine. The child has not yet received measles vaccine but has rfeceived other vaccines for children aged < 1 year. The child has no contraindications. Only two doses from the first vial have been administered since 8:30am, when the first dose was administered. Which of the two vaccine vials in the cold box would you use to vaccinate this child?

1. I would use the first open vial to prevent vaccine wastage ☐
2. I would tell the mother to return the next day, since I cannot open a new vaccine vial and there are no more children to vaccinate ☐
3. I would open the second vial of measles vaccine to immunize the girl ☐
4. I would recommend that the mother take the child to another health center to be vaccinated ☐
5. None of the above ☐

27. What instructions do you USUALLY give to caregivers the first time you issue them a new vaccination card?

(PLEASE CHECK ALL THAT APPLY)

1. Keep the card safe ☐
2. Bring this card to all visits to the health facility ☐
3. Bring this card only when you come for vaccinations ☐
4. No instructions are given ☐
5. Others: Specify: ___________________________ ☐

28. What do you do for a caregiver that forgot the vaccination card/health passport at home:

1. I do not vaccinate the child and ask mother to return with card next time ☐
2. I issue a new card, vaccinate and record today's vaccinations ☐
3. I issue a new card, vaccinate and record old vaccinations from the register ☐
4. I issue a temporary card, vaccinate and ask them to bring the old card for the next visit ☐
5. I will vaccinate without the replacing card, but I will document in register only ☐
6. Other: Specify: ___________________________ ☐

29. If a caregiver reports that the child's card has been lost or damaged, what do you usually do?

1. I issue a new card and record all future vaccines in the new card ☐
2. I issue a new card and transcribe all previous vaccines from register ☐
3. I issue a new card and ask woman to tell me of all previous vaccinations so I can write them down ☐
4. Vaccinate without replacing card, document in register only ☐
5. Other: Specify: ___________________________ ☐

IN THE FOLLOWING SECTION, DO YOU AGREE OR DISAGREE WITH THE FOLLOWING STATEMENTS:

30. Today, I have all the materials that I need to vaccinate patients who seek immunization (including syringes, recording sheets, vaccination cards/health passports, and other materials)

1. Agree ☐
2. Disagree ☐
   1. Vaccines ☐
   2. Syringes ☐
   3. Recording materials ☐
   4. Vaccination cards/health passports ☐
   5. Other ☐ _______________________

31. When the professional in charge of vaccination is unavoidably absent, a health care professional is available to replace him or her

1. Agree ☐
2. Disagree ☐

ADDITIONAL COMMENTS:

____

____
