# Supplementary material for: Assessment of missed opportunities for vaccination in Kenyan health facilities, 2016
Source: PLoS One. 2020 Aug 20;15(8):e0237913. doi: 10.1371/journal.pone.0237913 (PMC7440639; doi:10.1371/journal.pone.0237913)
Supplement: Attachment — Submitted filename: Response to Reviewers_.docx [file pone.0237913.s004.docx]

| **Assessment of missed opportunities for vaccination in Kenyan health facilities, 2016** | | |
| --- | --- | --- |
| **#** | **Reviewer Comments** | **Author Response** |
| **Editor Comments** | | |
| 1 | Please ensure that your manuscript meets PLOS ONE's style requirements, including those for file naming. | We have updated the manuscript and accompanying documents to comply with PLOS ONE’s style and file naming requirements. |
| 2 | Please provide additional details regarding participant consent.   a) In the ethics statement in the Methods and online submission information, please ensure that you have specified whether consent was written or verbal/oral. b) If consent was verbal/oral, please specify: a) whether the ethics committee approved the verbal/oral consent procedure, b) why written consent could not be obtained, and c) how verbal/oral consent was recorded. c) Since your study included minors under age 18, please state whether you obtained consent from parents or guardians in these cases. d) Please also indicate in your Ethics Statement whether all data were anonymised before the study authors accessed them. | a) We have included in the ethics statement in the Methods and online submission information that we obtained **verbal** consent from all participants. b) We followed the advisement of the Ministry of Health Kenya, which advised verbal consent over written consent, as written consent was not necessary for this program evaluation. The verbal consent procedure was approved by the Ministry of Health Kenya and was recorded by the interviewer on the data collection tool. c) Our program evaluation included interviews with adults (caregivers and health workers). Caregivers who were 15 years or older were included. This age cut-off was advised by the Ministry of Health Kenya and there was no additional informed consent obtained from these caregiver’s parents or guardians in these cases based on their advisement. d) We have included that no personal identifiers were collected. |
| 3 | Please note that all PLOS journals ask authors to adhere to our policies for sharing of data and materials: <https://journals.plos.org/plosone/s/data-availability>. According to PLOS ONE’s Data Availability policy, we require that the minimal dataset underlying results reported in the submission must be made immediately and freely available at the time of publication. As such, please remove any instances of 'unpublished data' or 'data not shown' in your manuscript and replace these with either the relevant data (in the form of additional figures, tables or descriptive text, as appropriate), a citation to where the data can be found, or remove altogether any statements supported by data not presented in the manuscript. | We have removed all instances of ‘data not shown’ and added the data to the appropriate tables or included descriptive text. |
| 4 | We note that you have included the phrase “data not shown” in your manuscript. Unfortunately, this does not meet our data sharing requirements. PLOS does not permit references to inaccessible data. We require that authors provide all relevant data within the paper, Supporting Information files, or in an acceptable, public repository. Please add a citation to support this phrase or upload the data that corresponds with these findings to a stable repository (such as Figshare or Dryad) and provide and URLs, DOIs, or accession numbers that may be used to access these data. Or, if the data are not a core part of the research being presented in your study, we ask that you remove the phrase that refers to these data. | We have removed all instances of ‘data not shown’ and added the data to the appropriate tables or included descriptive text. |
| **Reviewer #1** | | |
| 5 | The introduction and discussion can use a better framing of the purpose and implication of quantifying the missed opportunities. It seems a bit obvious that all contacts that did not result in vaccination if eligible will be a missed opportunity. Similarly, if health workers were not instructed to provide vaccination at every contacts that were not primarily for reasons of vaccinations, it is strange to ding them for not doing so.  Also it will help to give some context to the quantified missed opportunities. How does this compare to other countries? How bad is this? There was a quick description of the missed opportunities being much more if taking into account those without documentation of vaccination dates, but more can be said to further provide context to help readers understand the gravity of your results | Thank you for your review.  We have added information to the background to better frame the purpose and implications of quantifying MOVs in addition to context to quantify MOVs in Kenya in relation global figures.  Please note that the Kenyan national immunization guideline stipulates that every health service encounter should be used as an opportunity to provide missing vaccines. While some health workers may not have received this training, our assessment was aimed exactly at measuring the level of compliance to this national policy, which has been in place since 2013. |
| **Reviewer #2** | | |
| 6 | The authors have provided a good documentation of findings regarding missed opportunities for simultaneous vaccines. The methods for data collection and analysis are described reasonably, such that readers can readily understand the basis and organization of results, without delving unnecessarily deeply into statistical methods. As such this study provides a good foundational work of reporting as a benchmark for future studies. | Thank you for acknowledging our contribution to this body of knowledge. |
|  |  |  |
| 7 | To improve this study the authors might consider discussing the results in the context of specific action, rather than as a more general reporting of data. For example, the authors state that one in five surveyed health workers were unable to identify all of the vaccines in the national schedule. It could be additionally useful to know if there were specific vaccines or points in the recommended vaccination schedule that could be a point of focus for improvement. | We have added more details to the action items and recommendations to make them more specific and have ensured that they tie back to the data presented in the results. Other specific action-items are also listed in the final paragraph of the discussion, which outlines the action plan to reduce MOV as endorsed by the Kenya Ministry of Health based on findings from this MOV assessment. |
| 8 | Similarly, cross-tabulation of demographic variables against timeliness and MOV data cold provide additional insight into specific characteristics that tend to favor missed opportunities or late vaccines - or indeed those characteristics that favor no missed opportunities and consistently timely vaccines. | We have presented cross-tabulations of MOV by age (<12 months and ≥12 months) and reason for visit (Table 3) as well as timeliness of vaccines (Table 4). We chose to focus on these results for this paper as this data is linked directly to the actionable items endorsed by the Kenya Ministry of Health to reduce MOVs. We agree that additional cross-tabulations can give further insight into specific characteristics that may favor MOV (please see Annex 1). However, we have elected to exclude these from the manuscript as the MOV strategy is intended to address broad factors that are relatively easy to correct, but which could result in measurable improvements in vaccination coverage, as opposed to focusing on specific individual-level factors. |
| 9 | And, adding a statistical measure of significance, a "p" value, would provide to the reader a distinction between general reporting and significant findings. | We have omitted including statistical measures of significance such as “p” values or confidence intervals as this was not a random sample. Instead, we have attempted to highlight the most important findings in the discussion. |

**Annex 1. Missed opportunities for vaccination by demographic and other variables**

| **Variable** | **Total children with documented vaccination dates** | **Number of children needing 1+ eligible due doses** | **Missed opportunity for vaccination^1^** | |
| --- | --- | --- | --- | --- |
|  | N | n | n | % |
| **Child’s sex** |  |  |  |  |
| Male | 286 | 173 | 115 | 66 |
| Female | 264 | 167 | 127 | 76 |
| **Caregiver's educational level** |  | | | |
| None | 42 | 28 | 24 | 86 |
| At least some primary | 308 | 202 | 147 | 73 |
| At least some secondary | 204 | 109 | 71 | 65 |
| **Seen/heard vaccination messages in last month** | | | | |
| Yes | 3 | 78 | 57 | 73 |
| No | 426 | 260 | 184 | 71 |
| **Does your child have a mother-and-child health (MCH) booklet?** | | | | |
| Yes, and it is available at this visit | 509 | 307 | 212 | 69 |
| Yes, but not available at today’s visit | 38 | 30 | 27 | 90 |
| No | 9 | 4 | 4 | 100 |
| **Did staff ask for the MCH booklet?** | | | | |
| Yes | 426 | 269 | 176 | 65 |
| No | 129 | 72 | 67 | 93 |

^1^Among children needing 1+ eligible due doses
